# Healthcare professionals’ perspectives on medicine prescribing, vaccination, and alternative therapies in pregnancy and breastfeeding: A qualitative study from Catalonia, Spain

**DOI:** 10.1371/journal.pone.0345521

**Published:** 2026-04-29

**Authors:** Berta Munné-Barellas, Andrea García-Egea, Maria Giner-Soriano, Cristina Vedia-Urgell, Cristina Martínez-Bueno, Cristina Aguilera, Lina Camacho-Arteaga, Marta Lestón-Vázquez, Ainhoa Gómez Lumbreras, Laura Medina-Perucha

**Affiliations:** 1 Fundació Institut Universitari per a la recerca a l’Atenció Primària de Salut Jordi Gol i Gurina (IDIAPJGol), Barcelona, Spain; 2 Universitat Autònoma de Barcelona, Bellaterra (Cerdanyola del Vallès), Spain; 3 Unitat de farmàcia, Gerència Barcelonès Nord i Maresme, Institut Català de la Salut, Badalona, Spain; 4 Servei d’Atenció a la Salut Sexual i Reproductiva (ASSIR), Direcció Assistencial d’Atenció Primària, Institut Català de la Salut, Barcelona, Spain; 5 Sexual and Reproductive Health Care Research Group (GRASSIR), Barcelona, Spain; 6 Facultat d’Infermeria, Universitat de Barcelona, Barcelona, Spain; 7 Department of Clinical Pharmacology, University Hospital Vall d’Hebron, Barcelona, Spain; 8 Department of Pharmacology, Therapeutics and Toxicology, Universitat Autònoma de Barcelona, Bellaterra, Spain; 9 Àrea del Medicament i Servei de Farmàcia, Gerència d’Atenció Primària Barcelona Ciutat, Institut Català de la Salut, Barcelona, Spain; 10 Department of Pharmacotherapy, College of Pharmacy, University of Utah, Salt Lake City, Utah, United States of America; 11 Network for Research on Chronicity, Primary Care, and Health Promotion (RICAPPS), Madrid, Spain; All India Institute of Medical Sciences, INDIA

## Abstract

**Introduction:**

Healthcare professionals (HCP) encounter a multitude of challenges regarding the utilisation of medicines, vaccines, and alternative therapies in pregnant and breastfeeding women (PBW).

**Objective:**

The objective of this study was to explore the experiences, beliefs, and attitudes of HCP toward prescribing medicines, vaccines, and alternative therapies during pregnancy and breastfeeding in Catalonia, Spain.

**Methods:**

This study employed a qualitative methodology, guided by a gender-based perspective. Three discussion groups were conducted, with the participation of 21 HCP during February 2024. Sampling was intentional, purposive designed to ensure discourse diversity. Recruitment was conducted through key contacts in public primary healthcare centres, sexual and reproductive health care centres, hospital settings in Catalonia, using snowball techniques. The data were analysed using thematic analysis.

**Results:**

The challenges encountered by HCP who provide care to PBW are numerous and vary depending on the healthcare speciality area. Family physicians and nurses identified a lack of training in obstetrics as a factor contributing to difficulties in prescribing medicines. Midwives emphasised the importance of monitoring and follow-up during pregnancy, especially in cases where an adaptation of medication is required, while gynaecologists drew attention to the challenges posed by an increase in medication avoidance. HCP expressed concerns about the safety of the COVID-19 vaccine, as opposed to the greater safety of evidence of pertussis and influenza vaccines. The use of alternative therapies may lead to potential complications during consultations, as some users do not disclose their use.

**Conclusions:**

This study underscores the challenges encountered by HCP in drug prescriptions, complementary medicine, and vaccination during pregnancy and breastfeeding, considering HCP differences between specialities. Additionally, the study emphasises the difficulties associated with dual follow-up cases and the significance of establishing trusting relationships with users and shared decision-making processes or a good medication adherence.

**Patient or Public Contribution:**

HCP have participated sharing their experience in prescribing medication and recommendations on vaccines and alternative medicine. The results of this study can be used to effect change in the practice of HCP.

## Introduction

Medicines, vaccines, and alternative therapies (herbal remedies, homeopathy, acupuncture, aromatherapy, etc.) are commonly used during pregnancy and breastfeeding [1]. Healthcare professionals (HCP) visiting pregnant or breastfeeding women (PBW) often base their prescribing decisions on safety evaluations provided by the scientific community [2–4] while also combining different resources [4]. In 2015, the United States Food and Drug Administration published a new Pregnancy and Lactation Labelling Rule to guide medicines decisions during conception, pregnancy, and breastfeeding, a guideline also used in Europe [5]. Moreover, the European Medicines Agency requires the implementation of a Pregnancy Prevention Program aimed at minimising the risk of exposure to teratogenic or neurologically harmful medical products during pregnancy and conception [6,7]. In Catalonia, an interactive computer tool is employed to generate alerts and information for practitioners in primary healthcare. This tool identifies potential teratogenic risks, medical interactions, and other dimensions, thereby assisting in the treatment decision-making process [8]. Other tools are used by HCP in Spain, such as E-lactancia [9], a website that provides evidence-based information on the compatibility of medications and other substances during breastfeeding [10].

In recent years, there has been a tendency towards shared decision-making in healthcare consultations, is a model of patient-centred care that enables and encourages people to play a role in the medical decisions that affect their health [11]. Its aim is to reach an informed decision that is based on medical evidence but also reflects the user’s preferences. Some studies have reported that shared decision-making processes may help to reduce anxiety and increase knowledge and satisfaction among women [12]. Decision-making for HCP may appear to be straightforward, however, it ranges from acute treatments to managing chronic conditions) [2].

HCP recommendations, especially from midwives [13], are essential for vaccination [14–16] acceptance among PBW. Several vaccines are recommended during pregnancy in Catalonia, such as diphtheria, tetanus, pertussis, influenza and COVID-19 vaccines [17]. During breastfeeding, in addition to these, measles, mumps, rubella, and varicella vaccines are also recommended [18,19]. However, vaccination recommendations during pregnancy or breastfeeding should be personalised, assessing the risks and benefits in each individual case [20]. In Spain, some of them (e.g., influenza, pertussis) still have low coverage rates during pregnancy [21,22]. Building trust, and a good communication between HCP and users, and professionals’ knowledge and experience around vaccination are essential aspects to ensure vaccination, especially for those vaccines with low coverage [14,23]. Still, workload pressures and time constraints often prevent the effective delivery of these vaccines [23]. Moreover, some HCP feel poorly equipped to engage in difficult conversations with those who are reluctant/hesitant to be vaccinated [23].

Recently, the use of alternative therapies has increased, mainly among those seeking a more holistic and personalised approach and a preference to distance themselves from biomedical practices [24]. This trend is also linked to strains in public healthcare services (e.g., due to financial cuts), and in some HCP-user relationships [24]. In Spain, around 26% of the population have used alternative therapies at least once in their lives [25], although to our knowledge there are limited available descriptive data on the use of alternative therapies during pregnancy and breastfeeding. The lack of clinical guidelines and the limited knowledge of HCP about alternative therapies are considered the main barriers to effective and informed communication with users [26] about these options. For example, the protocol used in Catalonia to guide pregnancy follow-up lacks information on alternative therapies [17]. For these reasons, several studies have emphasised the need to promote the training of HCP in evidence-based alternative therapies to note that professionals can evaluate their recommendations and their subsequent integration into clinical practice, as well as at an institutional level and in future guidelines [26–30].

Differences between women’s health experiences and expectations and those of HCP, sometimes create barriers to build a trusting relationship. For example, it is important to consider the impact that the medicalisation and technification of pregnancy and childbirth [31–33] have had on medication use and how these practices have modulated relationships between PBW and HCP. As stated previously, HCP are faced with demands for which they often lack sufficient resources [32], often resulting in difficulties enhancing relationships with PBW [34]. Besides, in recent years women have claimed their agency in their own health processes [35]. This demand is particularly related to the fact that experiences of PBW have been under-researched due to ethical limitations in clinical trials [36], and due to androcentric approaches to women’s health in healthcare and research [37].

Qualitative studies are essential to delve deeper into how the dialogues of HCP are generated and interwoven with those of PBW. Such studies can help identify elements of the health system that interact with PBW health [38], enhance shared decision-making processes, and consider the experiences and needs of HCP. It is also important to consider the diversity of healthcare specialties. HCP may encounter distinct challenges and needs related to medicines, alternative therapies and vaccines, depending on their speciality and training. Moreover, the beliefs, experiences, and perceptions of HCP regarding the use of medicines, vaccines, and alternative therapies during pregnancy and breastfeeding have been rarely explored in our context. For this reason, this study aimed to explore the experiences, beliefs, and attitudes of HCP towards prescribing medicines, vaccines, and alternative therapies during pregnancy and breastfeeding in Catalonia (Spain). Specifically, it seeks to understand how different HCP understand and manage pharmacological and non-pharmacological treatments during these stages, and how their perspectives may be shaped by their professional background and other sociodemographic factors [38].

## Materials and methods

### Study design

This qualitative study employed a gender-based perspective [39,40]. The gender perspective is of crucial importance in this study due to the historical under-research of women’s health processes [37,41], especially during pregnancy and breastfeeding [42]. In addition, gender inequities in healthcare settings need to be addressed, as these may influence how HCP conduct their practice and their decision-making process (e.g., around the use of medicines, alternative therapies and vaccination). Approaching our research from a gender perspective could lead to more equitable health care and contribute to the promotion of social equity and justice for PBW [43,44].

The quality and rigor of this research were ensured by using Lucy Yardley’s criteria [45,46]. Consolidated Criteria for Reporting Qualitative Research (COREQ) checklist was used to guide the research process (see S2 File).

### Participants

Participants were 21 HCP in public primary healthcare centres (PHC). The inclusion criteria were: 1) working as a health professional in a primary healthcare or hospital in Catalonia; 2) providing care to PBW; 3) being able to communicate in Spanish or Catalan. Most participants identified their gender as women (N = 20) and were between 25 and 68 years old. Professionals were midwives (N = 4), gynaecologists (N = 2), family physicians (N = 6), family nurses (N = 6), paediatricians (N = 2), and a paediatric nurse (N = 1). Most worked fulltime in PHC, while four of them also worked in hospital care. Four participants worked in a rural area in the north of Catalonia, six in the city of Barcelona and eleven in an urban region in the Barcelona metropolitan area. Participants’ sociodemographic characteristics are available in Table 1.

**Table 1:** Participants’ sociodemographic characteristics (N = 21).

Discussion group (DG) number	Participants’ ID	Gender	Age	Healthcare setting	Profession	Urban/rural area	Years of experience
1	S1	Woman	47	Primary healthcare	Family physician	Rural	19
	S2	Woman	68	Primary healthcare and hospital healthcare	Paediatrician	Rural	45
	S3	Woman	41	Hospital healthcare	Gynaecologist	Rural	17
	S4	Woman	35	Primary healthcare	Family physician	Rural	7
2	S5	Woman	33	Primary healthcare	Family nurse	Urban	4
	S6	Woman	36	Primary healthcare	Family physician	Urban	6
	S7	Man	32	Primary healthcare	Gynaecologist	Urban	8
	S8	Woman	45	Primary healthcare and hospital health care	Midwife	Urban	12
	S9	Woman	34	Primary healthcare	Midwife	Urban	11
	S10	Woman	44	Primary healthcare	Family physician	Urban	8
3	S11	Woman	46	Primary healthcare	Paediatrician	Urban	15
	S12	Woman	39	Primary healthcare	Family physician	Urban	17
	S13	Woman	37	Primary healthcare	Family nurse	Urban	16
	S14	Woman	54	Primary healthcare	Family physician	Urban	25
	S15	Woman	46	Primary healthcare	Family nurse	Urban	24
	S16	Woman	29	Primary healthcare and hospital healthcare	Midwife	Urban	7
	S17	Woman	37	Primary healthcare	Midwife	Urban	15
	S18	Woman	60	Primary healthcare	Paediatric nurse	Urban	30
	S19	Woman	34	Primary healthcare	Family nurse	Urban	7
	S20	Woman	25	Primary healthcare	Family nurse	Urban	3
	S21	Woman	–	Primary healthcare	Family nurse	Urban	–
					with an average of years of experience of 14.8 years (SD 10.0)		

### Sampling and recruitment

Sampling was purposive. Researchers were mindful to ensure discourse diversity by recruiting participants of different healthcare specialities (midwives, gynaecologists, family nurses, family physicians, paediatric nurses, paediatricians), areas of care (PHC; hospitals), years of healthcare experience and geographical contexts (rural area; urban area).

Participants were recruited through key contacts (midwifes, gynaecologists and research team members) in PHC, sexual and reproductive health care (ASSIR) and hospital settings in Catalonia. Snowball samples techniques were also used. These centres were located in three urban cities (holding around 46,000–1,600,000 inhabitants) and one rural town (holding around 3,600 inhabitants). The names of the participating cities and town have not been disclosed in this article to ensure confidentiality and anonymity. Some healthcare professionals were recruiters of other professionals that meet the inclusion criteria. Then they provided the contact details of potential participants to the research team, who were responsible for contacting them and giving more information on the study. All selected participants agreed to participate, but at last moment some of them could not participate because of a change in the schedule. Recruitment was especially challenging as it was done at a time when several HCP strikes were going on.

### Data collection

Three discussion groups with 21 HCP were conducted in February 2024. The discussion group technique was chosen to explore narrative experiences, stimulate discussions, and account for participants’ interactions and group dynamics. The first (N = 4) was conducted in a hospital located in a rural area in the north of Catalonia. The second (N = 6) was carried out in ASSIR in an urban city, and the third (N = 11) took place in a PHC in an urban city. Data saturation was identified in the second group and reached in the third group, responses and themes were consistently repeated among participants across groups, and during second group we identified some consistency that were confirmed in the third group.

A topic guide was developed by the research team (see S1 File). The topic guide included questions on 1) experiences with medicine prescribing and vaccination during pregnancy and breastfeeding, 2) beliefs, experiences, and the role of alternative therapies during pregnancy and breastfeeding, 3) the decision-making process with users, and 4) resources used to prescribe medicines and vaccines, and recommendations on alternative therapies.

The study participants were informed about the interviewers’ work field and their motivation for doing this research. Before starting the discussion groups, participants provided both verbal and written consent and completed a sociodemographic questionnaire (see Table 1). Field notes were also taken. The discussion groups were conducted by LMP (N = 3), while BMB (N = 2) and AGE (N = 2) acted as observers, taking field notes and supporting the moderation. There was no prior relationship between the facilitators (BMB, AGE, and LMP) and most participants. BMB had a prior relationship with one participant. This relationship was approached reflexively, acknowledging its potential influence while ensuring professional boundaries throughout data collection and analysis. The session followed a predefined discussion guide, and the session was led by LMP. All analytical decisions were made collectively, which supported transparency, rigour, and the trustworthiness of the findings. This is further explained in the discussion section. The discussion groups lasted between 68 and 93 minutes and were audio recorded. A reusable water bottle was given to all participants as a token of thanks.

Regarding the research team, BMB (MPH) has worked as a research assistant in qualitative methods for over a year and AGE (MPH) has over three years of experience in qualitative and mixed-methods research. CMB is a health professional specialised in women’s health and sexual and reproductive health assistance and research. MGS, CVU, LCA, CAM, MLV, AGL have a wide experience in pharmacology and pharmacoepidemiology with expertise in primary healthcare clinical and hospital pharmacology research. LCA, CAM are consultants for teratology consultations of clinical practice. LMP (PhD) has ten years of experience conducting qualitative and mixed-methods research. The research team is committed to conduct public health research from a social determinants of health and gender-based perspective.

### Data analysis

Thematic analysis was used to analyse the data [47]. This included: a) familiarisation with the data, b) coding process, c) generating initial themes, d) reviewing themes, e) defining and naming themes, f) writing the analysis. The three discussion groups were transcribed and anonymised by BMB. Then, two were coded and triangulated by AGE and BMB. The third was coded by BMB. AGE, LMP and BMB carried out two team meetings to share and consolidate the coding, to explore the potential themes and propose a preliminary thematic framework. Although LMP, AGE and BMB shared the same perspective, there were several discrepancies in the coding process that were discussed and resolved in several meetings. Then, another meeting was organised with the whole research team to discuss the findings and the preliminary thematic framework. Discrepances in data interpretation were also discussed, helped to rethink the preliminary thematic framework and enriched the analytic process. The discrepancies within the research team were attributed to the fact that the data were more exploratory than descriptive. Through these meetings, it was possible to achieve a clearer alignment with the study objectives and ultimately reach a consensus. The results were drafted to deepen the analysis and incorporate ideas that had not been previously considered. When the draft was shared with the rest of the research team, changes were made to further deepen and finalise data analysis. A further meeting between BMB, AGE, and LMP was held to generate an organisation of data, so that the results were more in line with the aim of the study and the assessments of the other research members. These changes were incorporated into the final article and reviewed by all the members of the research team. Atlas.ti software was used for coding and data triangulation.

### Ethics approval and consent to participate

The study was approved by the ethics committee of the Institut de Recerca en Atenció Primària Jordi Gol i Gurina (IDIAPJGol) on 26^th^ Oct 2022, Ref 22/195-P. All activities included in the study were carried out according to existing guidance in ethics as indicated in the Universal Declaration on Bioethics and Human Rights adopted by UNESCO (19/10/2005); the Council of Europe Convention for the Protection of Human Rights and Dignity of the Human Being with regard to the Application of Biology and Medicine (1997) and its additional protocol on biomedical research (2005); the Helsinki Declaration (2013) and relevant EU laws (European Parliament and Council Directive 2001/20/EC); the Spanish Law on Biomedical Research (14/2007) and the LOPD (Spanish Law on Personal Data Protection) (3/2018). Participants received enough information to make an informed decision on participation. Identifiable data were deleted from the transcripts so that participants cannot be identified. All participants gave their informed consent to participate in the study.

## Results

Three themes were identified: a) Challenges for medicine prescription, adherence and monitoring; b) The lack of scientific evidence, the role of alternative therapies and new sources of information; c) The difficulties arising from double monitoring and the commodification of healthcare. The analysis was inductive and exploratory, and while some findings extended beyond the original descriptive scope, the identified categories and themes remained closely aligned with the main objective: to explore healthcare professionals’ experiences, beliefs, and attitudes regarding the prescription of medicines, vaccines, and alternative therapies during pregnancy and breastfeeding in Catalonia.

### Challenges in medicine prescription, adherence and monitoring

The participants largely concurred that the prescription of medication is a challenging aspect of their practice, especially during pregnancy but also during breastfeeding.

*“Yes, in pregnancy... there is no safe medicine, right? that is, you have paracetamol, but it is not either, you have two or three for the most common and the rest, every time, you use it, you consult it”* (S3, gynaecologist, GD1).

Many participants observed that PBW in general were reluctant to use prescribed medicines and it was uncommon for PBW to start any medication without first consulting.

*“It is true that patients in general are almost always very reluctant, which is very good, especially almost never does anyone start taking anything without having consulted them first”* (S3, gynaecologist, GD1).

S1, who was a family physician, commented that she typically preferred to wait for the “*natural healing progression*” before prescribing medication as it could help to prevent over-medicalisation. Family physicians often sought to build trust in their prescription decisions by discussing medication adjustments with gynaecologists or midwives.

*“It’s not what we do every day, you don’t have it so immediate, but... I think that even sometimes, maybe you give something less, right?”* (S10, family physician, GD2).

Additionally, family physicians observed instances in which PBW did not disclose their pregnancy status during medical appointments, which may have resulted in inappropriate medication prescriptions.

*“We as family physicians see pregnant women, but many times they don’t tell you that they are pregnant. And you have this perception of making prescriptions without knowing if she is pregnant or not (…) and the same in breastfeeding, (…)”* (S14, family physician, GD3).

Family physicians and nurses identified a significant challenge in the lack of knowledge about medication and the possible combinations needed when caring for pregnant patients with chronic conditions. Consequently, participants identified a need for training in obstetrics to provide more effective support and ensure greater confidence when prescribing. Other participants commented the potential value of having a clinical pharmacologist/pharmacist in PHC to address concerns towards medication use. S7, a gynaecologist, posited that family physicians are faced with a much wider array of pharmaceuticals because they treat a broader spectrum of conditions and typically manage patients who are on multiple medications. In contrast, midwives and gynaecologists reported that, in general, they had fewer doubts about the pharmacological decisions of their familiarity with pregnancy and breastfeeding. Particularly in the context of breastfeeding, several participants noted that the number of medications that can be prescribed is greater than during pregnancy because there are fewer contraindications which generates fewer concerns.

*“It is not like family medicine that the list of drugs that can be prescribed is endless. Of course, the pregnant woman is usually at most, 45 years old (…). The prescription is much simpler. But because we are trained in this from the beginning, I mean, our prescription catalogue is very short and very concise”* (S7, gynaecologist, GD2).

Participants reported that, in many cases, the follow-up of PBW living with a chronic or mental health condition was more manageable due to the involvement and follow-up of other HCP. Conversely, some professionals, especially family physicians, indicated that some PBW with chronic health conditions discontinued their medication without consultation, while others did not seek medical advice prior to becoming pregnant. As a result, these women either continued their previous medication during pregnancy or discontinued it without adequate follow-up. Participants explained that in a multitude of cases of PBW with chronic pathology, the cessation of medication could potentially exert a more deleterious impact on maternal health than on foetal development during gestation.

*“I think chronic pathologies it’s easier, because people usually prepare. In preconception visits, which is what people should do, plan the pregnancy, (…), in any type of pregnancy. And this with chronic pathologies is much simpler, they look for the right time, in general...”* (S16, midwife, GD3).

Some family physicians and nurses explained that the interdisciplinary exchanges of medical information were beneficial for them. Some participants even perceived the discussion group as a valuable space that facilitated knowledge exchange, and it was emphasised that effective coordination between HCP was essential to guarantee optimal follow-up. The role of midwives was considered significant, given their capacity to establish the most intimate and trusting relationships with users, facilitating adherence, promoting appropriate medication use and avoiding unnecessary apprehension.

Some participants, particularly those from urban areas, observed a lack of coordination between healthcare specialties. In contrast, professionals from rural areas noted effective collaboration between HCP, they found it easier to share uncertainties about prescriptions and had a deeper understanding of the users and the local context.

*Here we are a very small team, we work very side by side, and the matron who has given you the talk assists you at the birth. Here’s the thing, it can’t be another matron because there is no other“* (S3, gynaecologist, GD1).

Some participants noted that some deficiencies in coordination between specialities became evident during the postpartum period, as this was frequently the point of the transition from gynaecological to family care. Also, several participants observed that the computerised medical history sometimes lacked reliable data during pregnancy in cases where postpartum follow-up was unsuccessful or ceased. Participants commented that some PBW tend to prioritise the health of the infant in the postpartum period, which can result in a tendency to neglect their own health.

*“When they have given birth, I know they do check-ups, but they go all the way to primary healthcare and sometimes, they escape you. As they are with the child, they do not come to do the control analysis and tell you that they are tired, perhaps there is hyperthyroidism. I would say that perhaps a little more coordination is needed...”* (S6, family nurse, GD2).

The participants indicated that they typically relied on existing protocols when formulating prescriptions. They commented that protocols help to support decision making and facilitate the unification of criteria, particularly in relation to vaccination, which has significantly reduced user uncertainty. In addition, participants also consulted other resources: clinical guides (e.g., Medimecum [48]), and the E-lactancia [9] website. Participants highlighted the potential value of developing a digital resource similar to E-lactancia but focused on pregnancy.

*“We refer to the protocols, you recommend a flu vaccine and then you will see if it is effective or not. But we are linked to what they send us. In other words, we don’t have much option either to vaccinate those who are not in the protocol or to go around saying “don’t vaccinate” because there is supposed to be an advisory committee that we have to believe”* (S6, family physician, GD2).

In regard to vaccination, participants did not observe a significant amount of resistance among PBW overall. While influenza and pertussis vaccination are generally accepted by PBW, instances of vaccination refusal have increased. According to the participants, the historical overview of the influenza and pertussis vaccines provides a greater degree of certainty in recommending them because have proven to be effective. However, some participants expressed concerns about the methodology employed in the SARS-CoV-2 vaccine’s development, understanding the users’ reluctancies and its potential long-term public health consequences. Other participants emphasised that the vaccine is included in the protocols, therefore, should be recommended.

*“I think that the Covid-19 vaccine we can’t say anything for sure to anyone, because it’s really a different technique, it doesn’t look like any of the previous vaccines that we had used, and the manufacturing process didn’t go through the safety phases and everything, it was accelerated because it was needed, like that, right?”* (S1, family physician, GD1).

### The lack of scientific evidence, the role of alternative therapies and new sources of information

The participants highlighted the importance of accessing scientific evidence to make pharmacological decisions. Some participants asserted that their personal opinion should not be a factor in formulating recommendations for PBW. S17 observed that, sometimes recommendations based on scientific evidence did not effectively “reach the person”, which led to ongoing uncertainty. S7 stated that he relied on data and graphs (in accordance with the available scientific evidence) to address the typical doubts and reluctance of PBW, Despite this, participants identified significant barriers to prescribing, citing a lack of research and accessible resources. They pointed out that the available research is largely retrospective, as studying the pregnant population in a prospective manner is not feasible.

*“Of course, there is no [scientific evidence] because there is selection bias and the only thing, we have is retrospective data.”* (S7, gynaecologist, GD3).

Regarding alternative therapies, some participants expressed doubts about their efficacy suggesting that these therapies lacked sufficient evidence and could even be considered fraudulent. Nevertheless, other professionals acknowledged the growing interest and demand among some users. They noted that distinctions should be made between various therapies to determine which ones might be beneficial. For example, acupuncture or aromatherapy were perceived as useful techniques by S16, who highlighted that she had self-trained in aromatherapy and advocated for providing training opportunities to all professionals.

*“They did ask me what infusions I can take, because I had doubts about which ones could be taken (…) But there are some that are abortifacient. Well, I did a super bibliographic search and now I have infusions to drink once, some that can never be drunk, some that nothing happens. But I had to do it myself”* (S16, midwife, GD3).

S8 commented that neural therapy was a technique that was efficacious and could be offered in PHC. Both S1 and S8 emphasised the need for standardised training in alternative therapies, as they had to pursue their own in this area. Participants interested in alternative therapies noted that these practices are excluded from established protocols, although some admitted to using and recommending them. Some participants attributed their exclusion to two reasons: first, institutional resistance to these therapies within the health system due to the lack of evidence and secondly, the decision to introduce these therapies would depend on the willpower of each healthcare centre.

*“The [health service] is not in favour of alternative therapies, that’s why they also eliminated neural therapy that worked very well, for mastitis, for scars, for pelvic pain and suddenly it began to disappear little by little, we could no longer talk about neural therapy, we already had to talk about injections, right?”* (S8, family nurse, GD2).

A few participants suggested that identifying therapies supported by scientific evidence could help reduce medication costs and contribute to the development of a more efficient healthcare system. Particularly given the widespread perception that these therapies are harmless and the tendency of the population to use them without medical guidance, despite the potential risks involved.

Some participants highlighted the significance of social media as a conduit for health-related information, facilitating the dissemination of knowledge in a more equitable manner. S1, S14, and S16 acknowledged the importance of empowering PBW to make informed decisions regarding their own health. However, participants also explained that a considerable number of users arrived at consultations with specific demands.

*“I’ve been working for many years, before people came, listened to you and did what you told them. Which is not necessary either, a middle ground. But not now, now they come to tell you what to do. It’s different...”* (S14, family physician, GD3).

Some participants considered that these demands sometimes lacked coherence, yet but could not be necessarily rejected, as doing so would negatively impact on the relationship with the user. Conversely, some participants expressed concerns and frustrations when refuting PBW information obtained from “unreliable sources” (e.g., blogs, Instagram accounts run by mothers without training in gynaecology and obstetrics, doulas, journalists and psychologists).

*“Sometimes (…) you have to refute unfounded arguments from Google (…) because I’m a nurse, I’ve studied it and I’ve contrasted it with reliable funds and sometimes it’s very difficult because they’ve already looked at it before and they already know what they really have to do. They only come sometimes as a tool, don’t they? I want you to put this in my prescription or I need this”* (S5, family nurse, GD2).

Some participants observed that these changes are part of a paradigm shift about pregnancy, childbirth and breastfeeding. S17 explained that during the 1990s she saw a significant medicalisation of childbirth, whereas now there is a current tendency to de-medicalise it. Some participants commented that these previous practices have influenced the way women relate to the health system in the field of obstetrics, sometimes leading them to consult other specialists outside the biomedical system. S3, a gynaecologist, observed that gynaecologists are sometimes perceived as “aggressors”, especially during childbirth. Other participants expressed concern about a resurgence of traditional practices,. Moreover, S3 and S14 questioned whether “the natural childbirth movement had gone too far”, citing the resistance of PBW to certain interventions or the use of medicines, which generates difficulties in consultation.

*“Because it is true that there has been a very strong current for a few years, back to what was before, I don’t really know why, because, of course, what was natural before was that the mother or the child, or both, died, then it is true that we have many here, more than at the level of medicines, at the level of manoeuvres...”* (S3, gynaecologist, GD1).

### The difficulties arising from double monitoring and the commodification of health

Another significant issue that emerged from participants’ narratives, all of whom worked in the public system, was the challenge of double monitoring of pregnancy in both public and private health systems.

*“Of course, having double control, which I am always very against (..) there are two opinions, no one is right, and not because there are many opinions that are bad, but because of the confusion and because you create, in general, a... insecurity”* (S3, gynaecologist, GD1).

S14 explained that pregnancy is a period when more health insurance options are utilised, while S16 underscored the prevalence of health insurances in the context of Catalonia. The participants emphasized that the two health systems follow different logics, such as a greater number of diagnostic tests during pregnancy and a higher rate of caesarean sections, and a significant proportion also noted the use of outdated protocols in private healthcare centres.. S16 stated that in private healthcare, “you are not a patient, but a customer”.

*“And I think that the private sector plays a lot with it. All the ultrasounds so that you are as calm as possible, so that your uncertainty decreases”* (S4, family physician, GD1).

For instance, S12 highlighted the different approaches to medical practice, noting a proclivity among private healthcare professionals to request a greater number of diagnostic tests and a higher tendency towards caesarean sections..

Some participants indicated that they were opposed of double follow. Participants hypothesised that the practice of double monitoring results in a greater degree of uncertainty among users. The increased number of tests and controls conducted in private healthcare, along with the ability to perform all requested ultrasound scans, has led some users to perceive that the public health system is unable to provide adequate care and has a lack of resources.

*“Above all, it is to avoid the duplication of tests, because it only carries overdiagnosis, overtreatment and doubts, because each professional tells me something... Here, in the public test, since they don’t ask me for such a thing, it’s because they’re rats. Of course, I mean, there is a lot of mistrust. Then, I had to refuse some test”* (S7, gynaecologist, GD2).

Other participants noted that the private healthcare system provides a more extensive range of services, including access to in vitro fertilisation for individuals in older age groups who are not eligible for public protocols. S6 observed the current situation often prevents individuals from considering parenthood at the “biologically optimal time” due to the prevailing social context, which makes it challenging to achieve pregnancy. Furthermore, S6 questioned whether the protocol that defines pregnancy between the ages of 40 and 45 as high-risk should be updated.

Regarding alternative therapies and the involvement of other non-medical professionals such as doulas, some participants expressed concerns due to conflicts during consultations, like PBW refusing a prescription or vaccine in favour of alternative therapies. Additionally, the high cost of these therapies makes them inaccessible to all PBW, limiting recommendations based on affordability. In response, some participants proposed that integrating alternative therapies into the public healthcare system could ensure more equitable access.

Some participants highlighted the existence of territorial disparities in the accessibility of healthcare resources and social networks during pregnancy and breastfeeding. They explained that in some urban contexts, there was a noticeable decline in social and community networks which can affect the way users relate to the healthcare system. Several participants linked the increased reliance on private healthcare and alternative therapies during pregnancy and breastfeeding to the insufficient responses to women’s needs from the social context and public health services. They also acknowledged the resource limitations of the public healthcare system, which result in brief primary healthcare visits, frequent rotations of HCP, and a challenging environment for fostering trusting relationships between HCP and users. Such relationships are crucial for ensuring the appropriate prescription of medication and vaccines.

*“For me it is capitalism that makes the woman who has to give birth alone and work (…) and that there is no community,(…) you don’t know the neighbours. (...) if a pregnant woman doesn’t have any pathology, I don’t have to call her every week to see how she is, this is something that the society around her should do, of course here they find complementary medicine, they want someone to see her every week and tell her that everything is fine and that’s why they pay”* (S6, physician, GD2).

## Discussion

The participants encountered challenges in managing alternative therapies, citing a perceived lack of training in these therapies within their general practice. HCP considered a general acceptance among PBW to vaccination, but there has been an increase of the fears and insecurity towards the COVID-19 vaccine due to the dearth of knowledge available. Based on participants’ narratives, social media plays an important role in the dissemination of unfounded information, which have had a profound impact on the way the professional-user relationship is established. Besides, the participants have identified difficulties that arise from a dual pregnancy follow-up in the public and private healthcare settings. Furthermore, the prevalence of medical tests conducted in the private healthcare system has contributed to the perception among some PBW that the public system is deficient.

The findings of our study indicate that HCP face challenges when prescribing medication to PBW, particularly during pregnancy, as previous evidence has also shown [49], as there are significantly fewer contraindications during breastfeeding [50]. In Catalonia, family physicians and gynaecologists are the professionals responsible for managing medicine prescriptions. Family physicians highlighted the difficulties they encounter when prescribing medication, noting their relatively limited familiarity with the pregnant population compared to gynaecologists and midwives. Midwives were described as key references for PBW to build a trusting relationship throughout the pregnancy. The scarcity of scientific evidence on the compatibility of medicines with pregnancy is attributed to the ethical and legal constraints of conducting prospective studies involving pregnant and breastfeeding populations [49]. This generates challenges for HCP, especially in the context of chronic diseases, where medication administration can be vital during pregnancy [34].

As evidenced by previous literature and this study, a sense of caution is shared between HCP and women regarding the use of medication during pregnancy [51]. A fundamental aspect of obstetric care is the analysis of the risk-benefit ratio associated with the prescription of medications [2]. This decision is conducted by HCP with the aim of ensuring the well-being of both the mother and the foetus. All these concerns may influence women’s willingness to avoid medication, which could result in an exacerbation or a harm to the foetus [51]. Although caution is necessary, over-caution may limit the evidence that exists of pregnancy and breastfeeding, which may limit clinicians’ ability to provide effective care as they cannot make informed decisions, which has been previously explored [52].

In terms of vaccination, the manner of communicating information is of paramount importance, because the majority of individuals rely on HCP guidance to make informed decisions [53]. Vaccination schedules that have been established as safe and recommended during pregnancy in Catalonia include influenza, pertussis and SARS-CoV-2 [18]. Previous literature suggests that the SARS-CoV-2 vaccine has given rise to concerns among HCP due to the dearth of clinical trial data on its use in pregnant women, the uncertain risks to the foetus, and the prevalence of misinformation [54,55]. Despite endorsement in various protocols and recommendation from vaccination campaigns in Catalonia [19], our study revealed that a few HCP perceived a possible discrepancy between the recommendations from guidelines and their personal perspectives. This suggests the potential need for dedicated spaces that facilitate dialogues on how to balance existing scientific evidence with the practical and communication needs of HCP when conveying information to the public.

Some professionals have acknowledged the potential benefits of alternative therapies, yet due to the lack of empirical evidence, these are excluded from the protocols [56]. Conversely, others have deemed the majority of these therapies to be fraudulent. A common perception among the general public is that these products are safe and innocuous which often leads to results in women not disclosing their use to healthcare professionals during pregnancy [57]. This can be problematic as certain medicinal herbs have the potential to cause teratogenic effects [58]. There is a need to expand the evidence on these therapies and HCP are calling for an increase the scientific evidence and training in alternative therapies, given the rising prevalence of their use and the need for greater knowledge to ensure appropriate use [59].

Monitoring is of the utmost importance for reinforcing the HCP-user relationship, ensuring optimal adherence to medication regimes, and preventing the premature cessation of medication when it is required [12,38]. In Catalonia, the follow-up of pregnancies is managed by a variety of HCP, including obstetricians, midwives, family physicians and nurses. In the context of the public health system in Catalonia, midwives typically take on the role of closer monitoring during pregnancy, particularly for low-risk pregnancies [17,60]. This study and previous evidence have indicated that improved coordination and communication between professionals from different specialties can lead to enhanced health outcomes for the pregnant population [61]. In light of these findings, the participants’ narratives have highlighted the shortcomings of the Primary Care Clinic Station system in addressing the needs of professionals regarding prompt detection and alerts pertaining to pregnancies. It may, therefore, be of interest to consider the potential integration of the PREFASEG (Safe (SEG) Pharmacological (FA) Prescription (PRE) into Spanish) to the Primary Care Clinic Station system, an application designed to assist with the prescription of medications, analyse interactions between medicines and generate alerts for potential pharmacology harms [8]. It would be beneficial to explore the possibility of applying the functionalities of this tool to the specific case of pregnancy, with a view to resolving the conflicts faced by professionals [17].

In this regard, HCP occupy a crucial role in modulating users’ decisions. They play a pivotal role in ensuring optimal adherence to and follow-up of necessary medication and vaccination, as they are typically regarded as expert authorities [62]. It is therefore essential that HCP engage in shared decision-making with their patients, adopting a user-centred approach that fosters a sense of autonomy among users. This approach guarantees that patients are actively involved in the decision-making process, enhancing the quality of care provided to users [63]. This is particularly important given that the health system has been founded on the assumption that professionals are the primary sources of knowledge thereby that users are lacking in this regard [64]. It is necessary to engage in reflexive discourse concerning their social role and authority, particularly in an environment where more horizontal and participatory practices are being advocated [65]. In this sense, we identified some differences in the predisposition to implement shared decision-making among some participants. This points out to the need to continue advancing on the implementation of shared decision-making in professional training.

The individualism that characterises contemporary society has resulted in a reduction in the social support available [66]. Transformations in kinship structures, delayed conception, and the technological capacity to conception [33,67] have led to an increased demand for support from the healthcare system. Some of the HCP involved in our study stated that this level of accompaniment is not a necessary requirement during pregnancy in agreement with the Catalan Pregnancy guide [17] and that it should be provided by the social and family environment. Nevertheless, the need for more frequent follow-ups persists, and in cases where the PBW has the financial capacity, double monitoring is employed. In our context, 34% of users are covered by private health services [68]. This fact may be generating difficulties in consultations, as participants shared, since the follow-up by the public health system and private health follow different logics, unnecessary tests are promoted, and a client relationship with users is pursued [69]. Also, a participant highlighted that the commodification of alternative therapies, which are currently unregulated, represents a significant obstacle to their equitable access due to their exclusion from the public system. Other professionals operating outside the healthcare system (e.g., doulas) [61] may also be involved in pregnancy and breastfeeding. Some professionals have questioned the efficacy of doula accompaniment, citing concerns that these services may be commodified and perform tasks that midwives are already trained to do [70]. Conversely, these figures may play a social role in a context where some users have expressed a desire to de-medicalise obstetric processes.

Nevertheless, certain HCP have acknowledged that the health system has been particularly affected since the onset of the COVID-19 pandemic [69]. This situation has resulted in a shortage of personnel and the loss of community spaces due to the high turnover of professionals and the brief duration of their visits. This makes it challenging for HCP to establish connections with users and leads many individuals to seek second opinions and health follow-ups outside the public health system [71]. As some HCP have demanded that a community health approach should be applied to strengthen PHC and ensure the equity, efficiency and sustainability [72].

As evidenced in participants’ accounts, the processes of medicalisation of pregnancy and breastfeeding continue to influence the manner in which individuals engage with the health system [31]. The evolution of healthcare has been shaped by patriarchal structures, which have also resulted in the disregard of women’s health [41]. The practices carried out by HCP throughout the 1990s and the early 2000s and the medicalisation of bodies have been the subject of criticism [31]. Participants in our study and previous evidence have acknowledged that more rights are now being upheld and that some calls for de-medicalisation need to be balanced with the need for intervention [62,73].

In recent decades, the internet has become a primary source of information, alongside a plethora of questionable news that has proliferated. In the context of pregnancy and breastfeeding, social media networks have facilitated the exchange of experiences and needs among individuals [74]. However, it has also facilitated the dissemination of content and information that is frequently not subjected to critical analysis and may contravene the recommendations of HCP [75]. It would be interesting if HCP could inform PBW and their partners or companions about reliable sources of information that could help them to be informed.

### Strengths and limitations

This study contributes to advancing knowledge on the difficulties encountered by HCP when it comes to prescribing medication, vaccination and alternative therapies during pregnancy and breastfeeding within our healthcare context. Qualitative methodology provides valuable insights to the field of pharmacological studies, particularly when a gender-based perspective is employed. One of its strengths is considering the differences among healthcare specialties, in relation to prescribing, monitoring and the lack of information. Participants perceived the discussion group as a valuable space that facilitated knowledge exchange across different specialties. Moreover, this study incorporates the experiences of professionals from a range of territorial areas within Catalonia, thereby providing insight into the nuances of big cities and smaller rural villages.

Regarding the limitations, the research team faced considerable obstacles in assembling the discussion groups due to health strikes during the recruitment process. Despite a reduction in the number of participants, the quality of the discourse enabled the achievement of findings that were consistent with the objectives. Moreover, the researchers were unable to convene a group of HCP at high obstetric risk, which would have been a valuable addition to the study’s objectives. With regard to the rural discussion group, the level of participation was relatively limited, which may have had an impact on the representativeness of this group. Conversely, there was a notable representation from family physicians and nurses, while the involvement of gynaecologists and midwives was less pronounced, suggesting that the findings may be more reflective of the experiences and needs of the former two professional groups. Additionally, there was a relatively low level of male participation, with only one male taking part, which may reflect a gender disparity in interest in participating in the study. This limited participation could also influence the results, particularly regarding aspects such as the implementation of shared decision-making and other forms of interaction with PBW, as men may approach these differently. This method may also amplify certain voices, which should be acknowledged in relation to gender and experience roles in this study.

## Conclusions

Further research is required on the topics of medication, alternative therapies and vaccines during pregnancy and breastfeeding, to facilitate the formulation of evidence-based recommendations. This study identifies the challenges faced by HCP in relation to the prescription of medication, alternative therapies and vaccination during pregnancy and breastfeeding. A particular challenge was identified in relation to prescribing during pregnancy, and the management of chronic diseases, especially amongst family physicians. It is important to facilitate development and implementation of training programmes and resources for HCP to manage pregnancy and breastfeeding, with a particular focus on specific scenarios such as those involving diverse chronic medical conditions. Additionally, improved coordination among healthcare teams is essential to prevent gaps in follow-up when postpartum women transition from gynaecological care to primary healthcare. The role of midwives is fundamental in providing support especially throughout pregnancy and during the postpartum period, particularly in the initiation of breastfeeding, as they offer evidence-based guidance and assist postpartum women in successfully initiating and sustaining it. In this regard, for matters related to medication, vaccination and the use of alternative therapies, it is important for HCP to integrate shared decision-making into their daily practice to improve adherence and enhance women’s experience of pregnancy and breastfeeding by reducing the fears and concerns. Furthermore, addressing the use of online resources and social media as sources of information is essential, as users will continue to consult these platforms. This is particularly relevant in relation to alternative therapies, given that many postpartum and breastfeeding women rely on these digital sources. For this reason, it is imperative to work on recommending reliable and accessible sources of information, enabling the population to stay informed in their daily lives beyond clinical consultations. Finally, it is necessary to acknowledge the limitations that HCPs face during consultations, including the lack of time and other resources, which can contribute to their burnout and affect the quality of care.

## Supporting information

S1 File Additional file 1 Discussion groups’ topic guide. (DOCX)

S2 File Additional file 2 COREQ Check List. (PDF)

## Abbreviations

PBW: Pregnant and breastfeed women
HCP: Healthcare providers
PHC: Primary Healthcare Centres
ASSIR: Sexual and Reproductive Health Centres.
