# Supplementary material for: Healthcare professionals’ perspectives on medicine prescribing, vaccination, and alternative therapies in pregnancy and breastfeeding: A qualitative study from Catalonia, Spain
Source: PLoS One. 2026 Apr 29;21(4):e0345521. doi: 10.1371/journal.pone.0345521 (PMC13127937; doi:10.1371/journal.pone.0345521)
Supplement: S1 File — (DOCX) [file pone.0345521.s001.docx]

**Additional file 1. Discussion groups’ topic guide**

| 1. **How do you manage the prescription/monitoring of the use of medication during pregnancy?**     1. How is it during breastfeeding?    2. Ho do you manage it from nursery?    3. And in relation to vaccination? 2. **How do your decisions vary depending on whether the person is pregnant or not?**     1. And in the case of breastfeeding?    2. And if the patient has an acute disease? And if the patient has a chronic disease?    3. How do these decisions depend on the trimester of the pregnancy?    4. How is if the pregnancy is in vitro or with another assisted reproduction technique? 3. **What do you think about pregnant women and those who breastfeed using complementary medicine?** 4. **What role does alternative therapies/treatments play in the health system?**     1. And in relation to pregnancy and breastfeeding? 5. **What experiences do you have with recommending alternative therapies/treatments to pregnant and breastfeeding women?**     1. Some professionals have told us that sometimes they would like to recommend alternative therapies, but they feel that they should not do because of their role as health professionals. Has this happened to you? 6. **What do you think when users totally trust in your recommendations?** 7. **And what do you think about the reluctance they may have in taking the recommended treatment or not following the guidelines?**     1. And about not getting vaccinated?    2. How do you manage the reluctance with them? 8. **How is the decision-making process with the medication guidelines with the users?**    1. And how do you see this in the case of vaccination? 9. **What resources (for example, updated information) do you have to deal with the doubts that may arise with the use of drugs, vaccines and alternative therapies/treatments?**    1. What resources would you needed? |
| --- |
